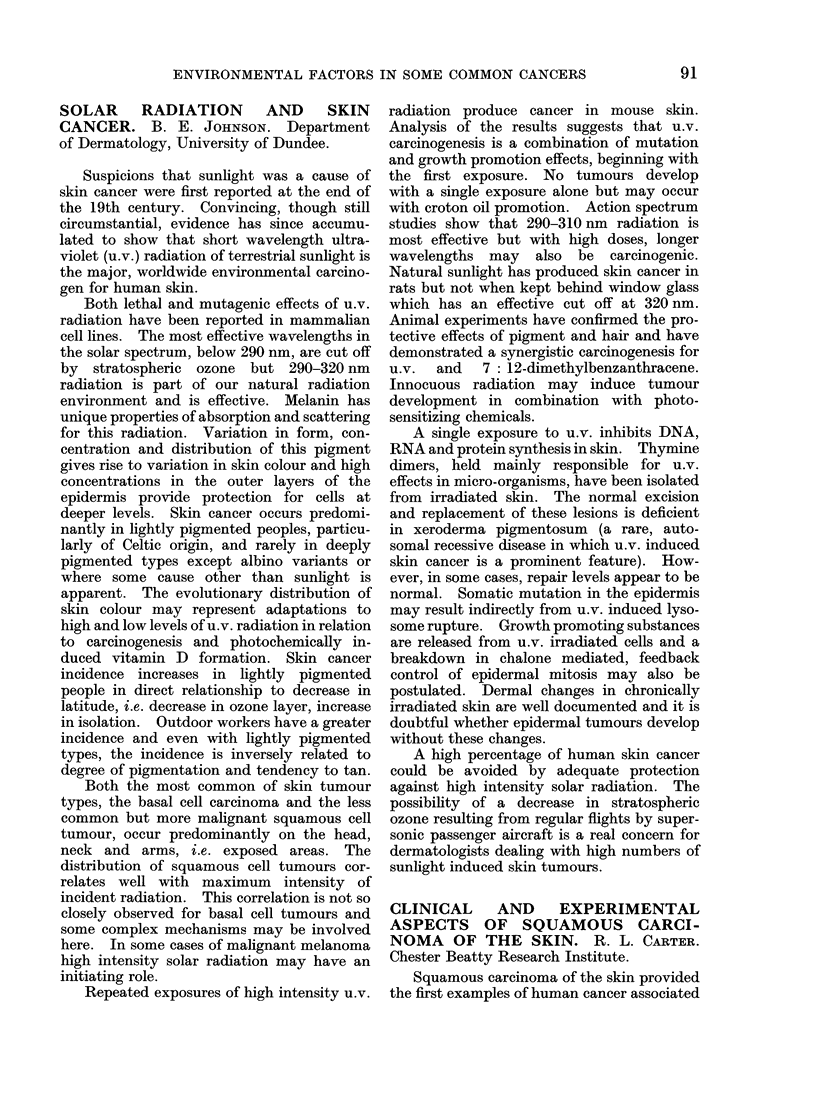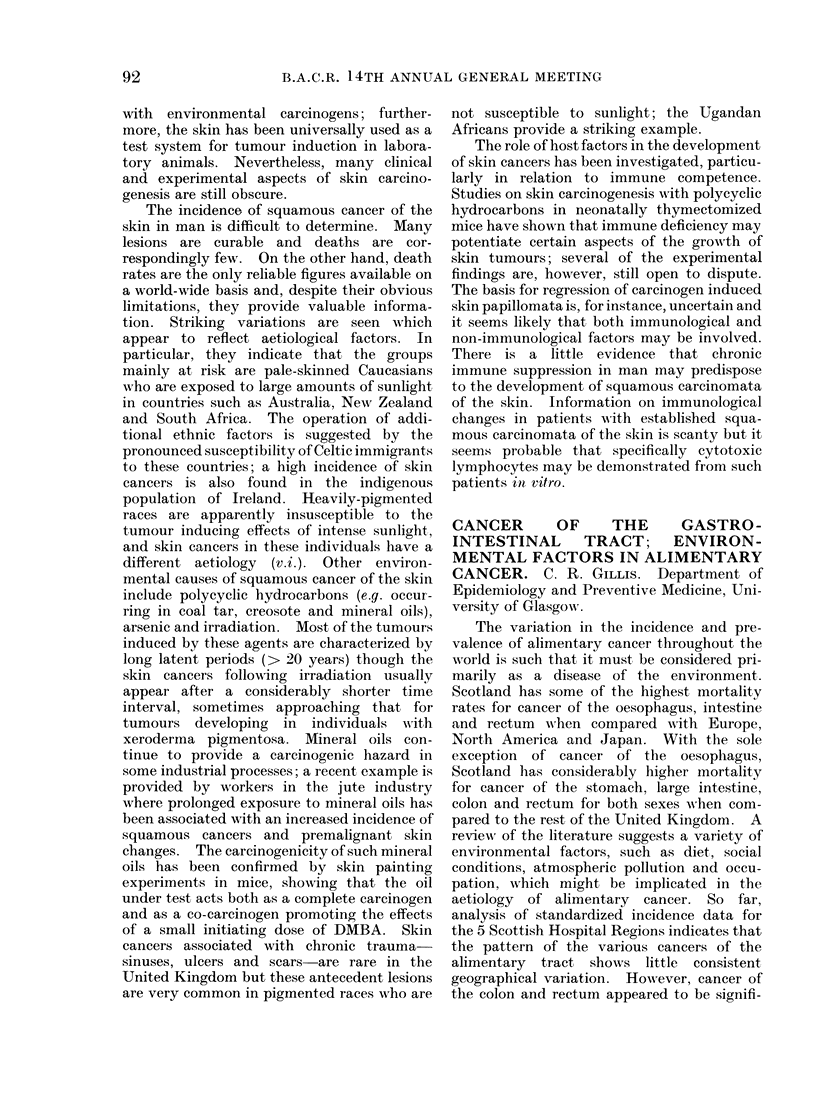# Clinical and experimental aspects of squamous carcinoma of the skin.

**DOI:** 10.1038/bjc.1973.119

**Published:** 1973-07

**Authors:** R. L. Carter


**CLINICAL AND EXPERIMENTAL ASPECTS OF SQUAMOUS CARCINOMA OF THE SKIN.** R. L. CARTER. Chester Beatty Research Institute.

Squamous carcinoma of the skin provided the first examples of human cancer associated with environmental carcinogens; furthermore, the skin has been universally used as a test system for tumour induction in laboratory animals. Nevertheless, many clinical and experimental aspects of skin carcinogenesis are still obscure.

The incidence of squamous cancer of the skin in man is difficult to determine. Many lesions are curable and deaths are correspondingly few. On the other hand, death rates are the only reliable figures available on a world-wide basis and, despite their obvious limitations, they provide valuable information. Striking variations are seen which appear to reflect aetiological factors. In particular, they indicate that the groups mainly at risk are pale-skinned Caucasians who are exposed to large amounts of sunlight in countries such as Australia, New Zealand and South Africa. The operation of additional ethnic factors is suggested by the pronounced susceptibility of Celtic immigrants to these countries; a high incidence of skin cancers is also found in the indigenous population of Ireland. Heavily-pigmented races are apparently insusceptible to the tumour inducing effects of intense sunlight, and skin cancers in these individuals have a different aetiology (*v.i.*). Other environmental causes of squamous cancer of the skin include polycyclic hydrocarbons (*e.g.* occurring in coal tar, creosote and mineral oils), arsenic and irradiation. Most of the tumours induced by these agents are characterized by long latent periods ($>$ 20 years) though the skin cancers following irradiation usually appear after a considerably shorter time interval, sometimes approaching that for tumours developing in individuals with xeroderma pigmentosa. Mineral oils continue to provide a carcinogenic hazard in some industrial processes; a recent example is provided by workers in the jute industry where prolonged exposure to mineral oils has been associated with an increased incidence of squamous cancers and premalignant skin changes. The carcinogenicity of such mineral oils has been confirmed by skin painting experiments in mice, showing that the oil under test acts both as a complete carcinogen and as a co-carcinogen promoting the effects of a small initiating dose of DMBA. Skin cancers associated with chronic trauma—sinuses, ulcers and scars—are rare in the United Kingdom but these antecedent lesions are very common in pigmented races who are not susceptible to sunlight; the Ugandan Africans provide a striking example.

The role of host factors in the development of skin cancers has been investigated, particularly in relation to immune competence. Studies on skin carcinogenesis with polycyclic hydrocarbons in neonatally thymectomized mice have shown that immune deficiency may potentiate certain aspects of the growth of skin tumours; several of the experimental findings are, however, still open to dispute. The basis for regression of carcinogen induced skin papillomata is, for instance, uncertain and it seems likely that both immunological and non-immunological factors may be involved. There is a little evidence that chronic immune suppression in man may predispose to the development of squamous carcinomata of the skin. Information on immunological changes in patients with established squamous carcinomata of the skin is scanty but it seems probable that specifically cytotoxic lymphocytes may be demonstrated from such patients *in vitro*.